# Encapsulation of Essential Oils in Nanocarriers for Active Food Packaging

**DOI:** 10.3390/foods11152337

**Published:** 2022-08-05

**Authors:** Shubham Sharma, Lilly Mulrey, Megan Byrne, Amit K. Jaiswal, Swarna Jaiswal

**Affiliations:** 1 School of Food Science and Environmental Health, Technological University Dublin—City Campus, Central Quad, Grangegorman, D07 ADY7 Dublin, Ireland; 2 Environmental Sustainability and Health Institute (ESHI), Technological University Dublin—City Campus, Grangegorman, D07 H6K8 Dublin, Ireland; 3 Centre for Research in Engineering and Surface Technology (CREST), FOCAS Institute, Technological University Dublin—City Campus, Kevin Street, D08 CKP1 Dublin, Ireland

**Keywords:** nanocarriers, essential oil, active packaging, antimicrobial, antioxidant

## Abstract

Active packaging improves a packaging system’s effectiveness by actively integrating additional components into the packaging material or the headspace around the packaging. Consumer demand and awareness have grown enough to replace chemical agents with natural active agents. Essential oils (EOs) are extensively distributed throughout nature but at low levels and sometimes with poor recovery yields, which poses an issue with their application in food. Due to the instability of EOs when added directly into a food product, they require encapsulation before being added to a packaging matrix such as liposomes, solid-lipid nanoparticles, nano-emulsions, cyclodextrins, and nanostructured lipid nano-carriers. This article is focused on the encapsulation of EOs in different types of nanocarriers. Nanocarriers can improve the efficiency of active substances by providing protection, stability, and controlled and targeted release. The advantages of the many types of nanocarriers that contain active substances that can be used to make antibacterial and antioxidant biopolymeric-based active packaging are discussed. A nanocarrier-encapsulated EO enables the controlled release of oil, stabilizing the packaging for a longer duration.

## 1. Introduction

Food packaging is known as a passive barrier that protects food from environmental factors like oxygen, water vapor, microbiological and chemical contaminants, pressure, ultraviolet light, and heat [1]. Food packaging has the main function of prolonging the shelf life of food. Food packaging also ensures that foods are transported and stored safely [1]. The primary goal of food packaging is to keep food in the most cost-effective way possible while also meeting industrial and consumer demands, ensuring food safety, and minimizing environmental impact [1]. The several primary functions of food packaging include preventing contamination or leaking, providing critical information about the food product contained within, such as nutritional value and cooking instructions, making purchasing a food product more convenient for consumers, and providing containment for ease of transportation and handling [2].

Innovative packaging such as active packaging, intelligent packaging, and smart packaging has grown in popularity and is frequently used in conjunction with packaging systems for foods, beverages, and pharmaceuticals [2]. The term “active packaging” refers to the incorporation of certain additives into packaging systems to preserve or expand product quality and shelf life [2]. Active packaging helps to reduce foodborne illness outbreaks and food recalls [3]. Intelligent packaging can be described as packaging that carries out intelligent functions such as monitoring the condition of packaged food, detecting changes in the state of the food and its surroundings (e.g., temperature and pH), and tracing, recording, and communicating these conditions [2,3]. Smart packaging is a packaging system that combines the functionality of intelligent and active packaging. It monitors the changes in the condition of the food product (intelligent packaging) and its environment, as well as acting on these changes (active packaging) [2].

Essential oils (EOs) have been recognized for their various biological activities for centuries. In ancient times, EOs were widely used in the preservation of foods, and possess some therapeutic properties, such as anti-septic properties for use on wounds and analgesic effects useful for numbing purposes such as clove oil. Currently, we still follow such practices in the domestic and industrial spheres, but they have been implemented in nature for far longer. Anti-parasitic activity has been observed against *Flagellate protozoa* when analysed with EOs [4]. To better understand the EOs’ potential for antimicrobial activity, they must be analysed individually, as their composition varies depending on many factors, meaning that the application potential for EOs is very extensive and can be a targeted area of research [5].

EOs are popularly known for their natural antioxidant properties that are increasing consumer demand due to the perception of their ‘safer’ natural origin. Antioxidants are of interest in both the food and pharmaceutical industries for their ability to neutralise free radicals and prevent oxidation which causes damage, producing negative effects. In foods, antioxidants are of use in preventing lipid peroxidation, which produces an off flavour and odour that negatively impacts the nutritional value and quality of the food, mainly in products with fats or oils. Within the body, antioxidants bind with free radicals and reactive oxygen and nitrogen species, which aid in the prevention of multiple diseases proven to be indirectly caused by free radicals, such as heart disease [6].

There are many issues and challenges with existing packaging, such as the use of plastic and environmental factors. Problems such as its non-biodegradable nature, recycling issues, and leaching of harmful chemicals into food lead to problems for the environment and human health [7]. Plastic, paper, glass, and metal are by far the most commonly used materials in existing food packaging. A broad range of plastics are used in food packaging [8]. Plasticizers, monomers, and oligomers found in packaging materials may be transferred to foods during the manufacturing process [8]. The transfer of chemical compounds between food and packaging is referred to as “migration”. The process of migration may cause changes in the quality and safety of the food product, as well as sensory changes due to the transfer of undesirable components from the packaging material to the food product [8].

This article aims to critically review the current state of the art in the encapsulation of EOs in nanocarriers for active food packaging applications. Various important aspects of EOs application in food packaging such as common nanocarriers for encapsulating EOs, migration of EOs from active packaging, current application, and legal aspects of the use of nanocarrier active agents in food packaging have been discussed.

## 2. Active Packaging

Active packaging is an emerging concept that has sparked a lot of interest and concern over the past decade or two. According to Yucel, (2016) active packaging can be described as a sophisticated packaging system with enhanced protection properties, such as both antioxidant and antimicrobial effects, with an improved capability greater than that obtained through conventional packaging methods [9]. The main aim of active packaging is to enhance the performance of a packaging system by purposely introducing subsidiary constituents in either the packaging material or the headspace surrounding the packaging [10]. This innovative system helps to ensure food quality and safety while also extending the shelf life of products [11]. It has the ability to replace the addition of active substances to foods, limit particle migration from packing materials to food, as well as eliminate industrial processes that can lead to the presence of pathogenic microbes in the product [3]. Active packaging includes ethylene absorbers, carbon dioxide emitters, antioxidant packaging, antimicrobial packaging, moisture absorbers, and O_2_ absorbers [11].

**Figure 1:**
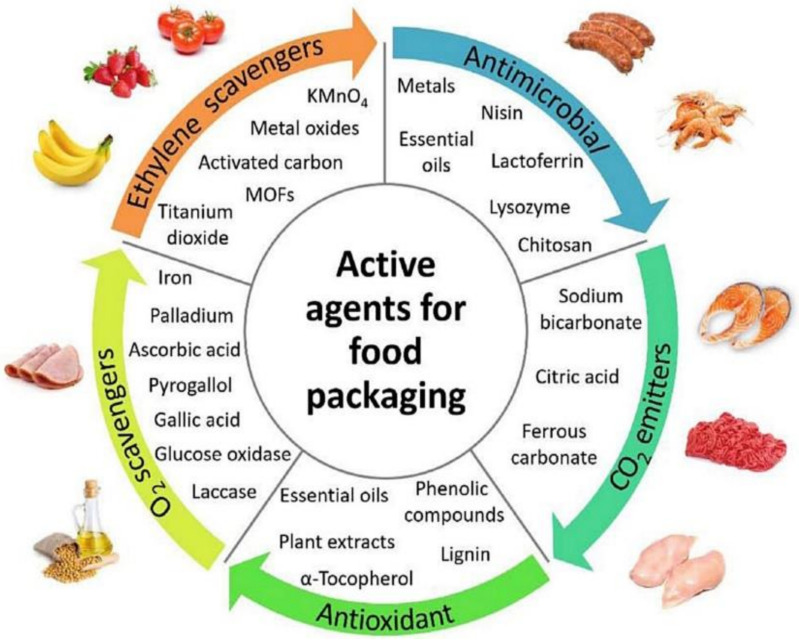
Active agents used in food packaging (Adopted from Vilela et al. [13]).

Active packaging can be created by implementing antimicrobial agents into the packaging composition [1]. The use of antimicrobial agents in packaging can create an environment within the food packaging that can delay or prevent the growth of microorganisms on the food product. This, in turn, increases the shelf life and safety of the food product [12]. Figure 1 concisely highlights the typical active agents used commercially in food packaging that are characterized as antimicrobial agents, antioxidant agents, oxygen scavengers, ethylene scavengers, and carbon dioxide emitters, and examples of each are given [13]. In more recent years, attention has turned to a more ‘natural’ approach to active packaging, as opposed to the current practice of using active packaging materials that may contain modified gases, dyes, and inorganic compounds. EOs, organic acids, enzymes, biopolymers, antimicrobial peptides, and organic acids are all antimicrobial agents [12,14]. Antioxidant packaging prevents unsuitable oxidation and aerobic microorganism growth. This type of packaging reduces the metabolism of the food product [11]. O_2_ absorbers lower the oxygen concentration to less than 0.01 percent, reducing O_2_ diffusion in food packaging [15]. O_2_ absorbers are made up of chemical substances, including iron, ascorbic acid, or enzymes, that can react with oxygen and absorb up to 2000 cc of O_2_ from 10,000 ccs of air [16]. Ethylene is a growth hormone that fruits and vegetables produce during storage. Ethylene increases food respiration, lowering the shelf life of food products.

Using moisture absorbers is an effective way to control excess moisture development inside food packaging. The moisture absorber controls the water activity of the food product to inhibit the growth of microorganisms. Moisture absorbers are commonly used in the form of sachets, films, trays, and pads to keep excess fluid from food products [17].

Chemo-active and bioactive packaging are two main types of active packaging [3]. In chemo packaging, chemicals are used as an active agent in the packaging; chemo packaging methods affect the chemical composition of the food item as well as the gaseous atmosphere within the packaging [3]. O_2_ in food packaging promotes the growth of bacteria, causing the food product to produce undesirable sensory characteristics. Different oxygen-reactive materials such as titanium, iron, and zinc can be used as oxygen scavengers in active packaging [18]. The use of some synthetic antioxidants in chemo-active packaging may have some drawbacks. For example, butylated hydroxy anisole, a synthetic antioxidant that protects against lipid oxidation and is used in chemical active packaging, may have a negative impact on an individual’s endocrine system [19,20]. Bioactive packaging involves antimicrobial compounds engaging with biological molecules like bacteria, and can influence biological processes, which in turn hinders the growth of several microorganisms [3,21]. The addition of antimicrobial compounds (such as EOs) into packaging films is an example of bioactive packaging [22]. Azadbakht et al. (2018), investigated the antimicrobial activity of packaged sliced sausages by integrating *Eucalyptus globulus* EO in chitosan. The results revealed that increasing the concentration of EO (*Eucalyptus globulus*) can enhance the log reduction value [23].

Active packaging is an innovative method that prolongs the shelf life of food; however, it has many advantages and disadvantages compared with chemical active packaging. For example, chemical active packaging may have negative health effects as well as render packaging unrecyclable, resulting in significant waste [3].

## 3. Essential Oils as an Active Agent

EOs are derived from aromatic plants and are classified as volatile liquids, commonly obtained through different processes such as solvent extraction, hydro-distillation, steam distillation, and hydro-diffusion [3,24,25]. An EO is defined as a ‘product obtained from a natural raw material of plant origin, by steam distillation, by mechanical processes from the epicarp of citrus fruits, or by dry distillation, after separation of the aqueous phase, if any, by physical processes’, according to the International Organization for Standardization (ISO) [3]. EOs vary greatly, partly due to genetic causes, as well as climate, rainfall, or geographic origin [24]. EOs consist of mainly lipophilic and highly volatile secondary plant metabolites, primarily monoterpenes and sesquiterpenes; however, other compounds like allyl and isoallyl phenols, along with alkaloids and coumarins, are also common. EOs include lavender (*Lavandula angustifolia*), peppermint (*Mentha piperita*), tea tree (*Melaleuca alternifolia*), clove (*Syzygium aromaticum*), sage (*Salvia officinalis*), cinnamon (*Cinnamomum zeylanicum*), eucalyptus (*Eucalyptus globulus*), lemongrass (*Cymbopogon citratus*), and *origanum vulgar* [26].

The composition of EOs varies from plant to plant and depends on the type of plant it is derived from, the environment in which it grows, and the part of the plant required to obtain the EO (stem/bud/leaf), etc. For this reason, EOs contain many properties of interest to consumers, producers, and medical practitioners with the potential to perform anticancer, anti-inflammatory, antimicrobial, antidepressant, antianxiety, antidiabetic, and many other beneficial biological functions [27]. Because of their hydrophobic nature and lower density than water, they are lipophilic, soluble in organic solvents, and insoluble in water [28]. The process of hydro-distillation involves submerging plant materials in a water-filled vessel and boiling the mixture. The main advantage of hydro-distillation is the extraction of hydrophobic plants with a high boiling point, and the technique enables the extraction of plant material at temperatures below 100 °C [3].

Steam distillation is the most common method for the commercial production of EOs [29]. In this process, the plant substance is heated with the use of steam. The steam can only pass through the plant while the boiling water does not mix with the plant substance. The heat supplied by the steam releases the EOs [3]. Hydro-diffusion extraction involves reducing the steam temperature to under 100 °C. The vacuum is provided by the top of the generator, which is kept at a low temperature. In this process, the dried plant materials and the steam are supplied in a container [3]. The solvent extraction method involves solvents such as hexane being mixed with the plant material and slightly heated and filtrated, the solvent eventually evaporating. To dissolve the EO, the final mixture is mixed with alcohol, and finally, at a low temperature, the distillation process occurs [3].

EO’s primary function is antimicrobial and antioxidant activity [1]. According to recent studies, EOs are natural preservatives and are recognized as fresh and safe chemical additives. Research findings have shown that high intakes of EOs can reduce the risk of various cancers as well as cardiovascular disease [26].

**Figure 2:**
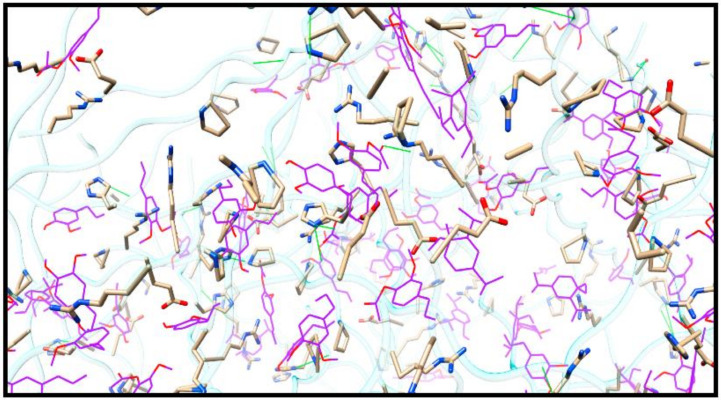
View of the EO-impregnated biofilm in greater detail, showing the side chains of the amino acids that interact with the EO molecules (colour purple). Sticks are used to symbolize the side chains of amino acids, while the wire is used to represent EO compounds. Hydrogen bonds are represented by the green lines. Atoms are represented by colour; for example, oxygen is red, and nitrogen is blue (Adopted from Cruz et al. [33]).

EOs are natural substances that have been shown to be effective in replacing synthetic food additives. They can be incorporated into food packaging by slowly releasing their compounds into the food product. Many essential oils derived from plants, such as basil (*Ocimum basilicum* L.), chamomile flowers (*Matricaria chamomilla* L.), and rosemary (*Rosmarinus officinalis* L.), have been used in food packaging. These essential oils used in food packaging are classified as GRAS [30]. Fernández-Pan et al. investigated the effectiveness of oregano and garlic EOs in immediate contact with chicken breast, and incorporated them into a protein matrix. After 13 days of storage, it was discovered that EOs controlled microbial action when applied directly to the chicken’s surface [31]. However, there was less adhesion of the antimicrobials on the chicken surface when compared to their use within a structural matrix. This meant that antimicrobials were distributed uniformly and remained at ineffective doses throughout the storage time [31,32]. Cruz et al. studied the bioactive compounds in *Piper divaricatum* EO, and the molecular interactions established with a biofilm. The gelatin matrix included small amounts of the main EO components. The polypeptide chain that makes up the biofilm was subject to non-covalent interactions by these substances. The biofilm’s structural, mechanical, and antioxidant characteristics changed as a result of oil impregnation. Additionally, EO changed the biofilm’s physical characteristics, reducing its traction resistance and boosting its ability to extend [33]. Researchers have also represented the chemical interactions of the hydrophobic nature, as can be seen in Figure 2.

## 4. Common Nanocarriers for Loading Antimicrobial Agents in Active Packaging

### 4.1. Nanoliposomes

Nanoliposomes are spherical-shaped lipid carrier vesicles of nanometric size (10^−9^) containing hydrophobic hydrocarbon tails of phospholipids that are arranged into a single or multi-concentric bilayer [34]. They are organized in a way that the polar head groups face the aqueous phase of both the inner and outer media of the environment they are present in. This feature allows the nanoliposomes to trap both hydrophilic compounds within the aqueous centre and the lipophilic compounds within the bilayer compartment, and results in the encapsulation of the compound. Nanoliposomes are said to be capable of encapsulating, delivering, and releasing water-soluble, lipid-soluble, and amphiphilic materials which makes them a useful tool for the food industry. Nanoliposomes that are used in the food industry are derived from natural sources that contain biologically active phospholipids, such as milk, egg, and soy. Nanoliposomes are widely used in active packaging as a suitable nanocarrier for particularly hydrophobic compounds, such as oil- or fat-based antimicrobial agents, and can be produced using non-toxic methods, such as heating and micro-fluidization.

Blanco-Padilla et al. describe the interactions in active food packaging between the nanoliposome and the external target cells [35]. This involves the adsorption onto the food surface and fusion with the cell membrane followed by the release of an active substance. They studied the encapsulation of nisin through a high-pressure homogenization method known as micro-fluidization, in lecithin from soybean; they also investigated the antimicrobial activity of nisin through transmission electron microscopy, which identifies the degradation of the nanoliposome through pore formation, causing the release of nisin externally. The nanoliposomes were also embedded in a hydroxypropyl methylcellulose (HPMC) matrix to allow for a slower, more controlled release of nisin, which proved to result in antimicrobial activity and inhibitory properties towards *L. monocytogenes*. Subramani and Ganapathyswamy classified the liposomes into five types according to the mechanisms of intracellular compounds [36], as follows:

1. Conventional liposomes: The lipid layer is negatively and positively charged. It also has phospholipids and cholesterol attached to the aqueous core. The lipid bilayer can penetrate through both hydrophobic and hydrophilic compounds;
2. pH-sensitive liposomes: The lipid composition of liposomes destabilizes once the external pH changes from neutral to an alkaline or acidic environment;
3. Cationic liposomes: The liposome contains a positively (cationic) charged phospholipid that interacts with negatively charged compounds and nucleic acids during a mixing process;
4. Immuno-liposomes: The liposome contains antibody molecules or substances within the liposomal surface;
5. Long circulating liposomes: The hydrophilic area of oligosaccharides, glycoproteins, polysaccharides, and synthetic polymers are coated with the liposome surface to assist the prolonged circulation of the liposomal material (mainly relevant in drug delivery systems).

Nanoliposomes provide a greater surface area which would proportionally increase the yield of adsorption allowing for an eco-friendlier approach toward active packaging [36]. Esmaeili et al. observed the encapsulation of essential garlic oil in a cholesterol and soybean phosphatidylcholine-based nanoliposome, embedded within a chitosan film, to produce antimicrobial and inhibitory effects. They demonstrated the retardation of lipid oxidation when compared to the control subject’s data based on stability analysis [37].

### 4.2. Solid Lipid Nanoparticles (SLNs) and Nanostructured Lipid Carriers (NLCs)

Solid Lipid Nanoparticles (SLNs) and Nanostructured Lipid Carriers (NLSs) are two novel approaches to delivering lipid-based compounds within a system, with food packaging applications in particular due to the biodegradable nature of lipids present, without the requirement of an organic solvent for their synthesis. The biocompatibility of the solid lipid nanocarriers makes them ideal for use in food packaging [38].

SLNs are oil-in-water nano-emulsions in which the liquid lipid (oil) has been replaced by a solid lipid (fat) or a blend of fats that remains solid at room temperature. These nano-vehicles were created to overcome the drawbacks of polymeric nanoparticles (use of organic solvents, unable to scale up), emulsions (uncontrolled rapid drug release), and liposomes (uncontrolled rapid drug release). According to Mehnert, SLNs have introduced new carrier systems with good stability, regulated release, the ability to shield sensitive drugs from degradation, and the possibility of large-scale manufacture [39].

Unlike an SLN, which is made up of solid lipids, an NLC is made up of a mixture of solid and liquid lipids. In comparison to an SLN, the solid lipid matrix formed using liquid lipid in an NLC minimizes drug leakage during storage, and improves drug entrapment and the release properties of NLCs [40]. A study carried out by McDaniel et al. investigated the anti-fungal properties of pullulan packaging systems loaded with EOs such as thymol, eugenol, and cinnamaldehyde, using both liquid (coconut oil) and solid (hydrogenated palm oil) carrier oils [41]. The oils were encapsulated to create liquid nanodroplets and solid-liquid nanoparticles, which produced anti-fungal effects when studied in vitro.

Souto and Severino reported on the use of NLCs and SLNs as encapsulating systems for antioxidants (such as carotenoids, Vitamin E, and tocopherols) with potential specifically for food packaging applications [42]. These are defined as colloidal systems (50–1000 nm) that can provide extensive physical and chemical stability, which would prevent the degradation of bioactive compounds, and capable of delayed or prolonged release within a food packaging system. Oils deemed suitable for use as an NLC in the food industry are palm, sunflower, soybean, and peanut. Alternatively, the solid lipids suitable for use as an SLN are carnauba wax, bees wax, and compritrol.

### 4.3. Cyclodextrin (CD) Nanocarriers

Cyclodextrins (CDs) are cyclic oligosaccharides in a truncated cone shape that are obtained through the enzymatic digestion of starch, classified into α-cyclodextrins, β-cyclodextrins, and -cyclodextrins based on the number of glucopyranose units, respectively 6, 7, and 8 [43]. The inner cavity of cyclodextrins is a hydrophobic region consisting of pyranose rings, whereas the outer surface obtains a hydrophilic nature due to the presence of hydroxyl groups of glucose units.

The structure of cyclodextrins is arranged so that the core of the cone is a hydrophobic region, and the outer shell is a hydrophilic region, allowing for a wide range of interactions between molecules and compounds that can form inclusion complexes, such as ions, oligonucleotides, and proteins. The amphiphilic nature of cyclodextrins is highly desirable in both the food and pharmaceutical industries, used as a solubility enhancer, particularly for low solubility bioactive molecules, which would in turn improve their stability. Cyclodextrins possess the ability to form non-covalent bonds with guest molecules, making them a potential nanocarrier for food packaging applications [44]. The article describes the bonding forces within cyclodextrins and guest molecules which can prevent and protect them from damaging processes like oxidation, degradation, and evaporation; such bonding forces between guest molecules and cyclodextrins include hydrogen bonds, dipole-dipole interactions, and van der Waals forces. Furthermore, it was reported that the inclusion complexes of bioactive molecules with cyclodextrins influence the release of bioactive compounds, with the stability of the inclusion complex dependent on the type of bonds present between cyclodextrins and their guest molecules [45].

Cyclodextrin’s abilities to act as a nanocarrier were further investigated by Chen and Liu, the authors focusing particularly on food packaging applications [46]. They studied the incorporation of β-cyclodextrin with mustard EO to create a cellulose sulphate film that would allow for a slow release of the active agent, in this case mustard EO. The presence of mustard EO in a film demonstrated successful antimicrobial activity against *E. coli*, *Staphylococcus aureus*, *Bacillus subtilis*, and *Aspergillus niger* (Table 1). Mustard oil incorporated with the β-cyclodextrin present in a film demonstrated more potential, as it indicated a slower release of the mustard oil when compared to that without a cyclodextrin. Cui et al. (2018) used tea tree EO paired with β-cyclodextrin to pose as a host guest, allowing for the formation of a water-soluble inclusion complex that was proven to contain antimicrobial properties against *E. coli* on a beef sample [47]. It was further discovered that by applying plasma treatment to the cyclodextrin tea tree oil complex, specifically cold nitrogen plasma treatment, there was a significant increase in the antimicrobial activity, as well as evidence of the more efficient release of the active or antimicrobial agent, indicating potential for application in food packaging.

### 4.4. Nanoemulsions

Nanoemulsions are known to be biphasic dispersions of two immiscible liquids: water in oil (W/O) droplets or oil in water (O/W) droplets, stabilized by an amphiphilic emulsifier [55]. They are common lipid-based encapsulation systems and are composed of two immiscible liquids (oil and water), which become stable by including suitable emulsifiers [1].

The emulsion system chosen is determined by the nature of the antimicrobial agents and the purpose of the encapsulation. An example of nanoemulsions used for hydrophilic antimicrobials would be W/O nanoemulsions; however, the lipophilic agents can be encapsulated in O/W nanoemulsions. O/W nanoemulsions are a suitable option for the encapsulation of antimicrobial EOs. In this instance, the EO is used as the oil phase, which is then dispersed into the water phase containing emulsifiers [1]. O/W nanoemulsions are composed of oil droplets, with an average droplet size of 20 to 200 nm that is distributed in an aqueous medium and stabilized by an emulsifier component [56].

Nanoencapsulation involves creating nanocarriers loaded with bioactive compounds that come in a variety of shapes, such as nanocube, nanocapsule, nanosphere, nanoshell, branched, nanostar, nanocluster, and nanorod [1]. The droplets typically have an average size of 50 to 500 nm, although they can be larger [57]. Due to their small droplet size, nanoemulsions tend to be transparent. This makes them a good candidate for use in the food and beverage industry [58]. Macroemulsions have a larger droplet size which is visible as an opaque colour [57]. Nanoemulsions also have a higher loading capacity for lipophilic drugs than microemulsions [59]. Other properties due to their small size include a high surface area per unit volume, higher stability, increased bioavailability of lipophilic components, and flexible fluidity [60].

They are commonly classified as nanoemulsions, nanoliposomes, nanofibers, nanohydrogels, and nanoparticles [26]. Nanoemulsions can be produced in a variety of dosage forms, including creams, gels, aerosols, liquids, sprays, foams, and gels. The distribution of nanoemulsions includes a multitude of methods including ocular, intranasal, intravenous, topical, and oral [51]. Nanoemulsions have a higher solubilization capacity than simple micellar dispersion and increased kinetic stability than coarse emulsions [51]. Nanoemulsions are used as an aqueous base for organic substances in the pesticide as well as food and beverage industries [51] via ultrasonic processes as well as high-pressure homogenization [1]. Nanoemulsions can be created using one of two methods:

(i) Top-down methods

These methods include high-pressure homogenization, ultrasonication, high shear homogenizer, micro fluidization, and membrane emulsification, and are used to break down the lipid phase into linearly sized droplets. These methods are also known as high-energy methods [26];

(ii) Bottom-up methods

These methods include emulsion phase inversion, solvent de-mixing, and spontaneous emulsification. Bottom-up methods are used to transform the assembly of molecular building blocks into effectively structured systems, as imposed by the thermodynamic equilibrium. These are known as low-energy methods [26].

### 4.5. Biopolymeric Nanocarriers

Biopolymers are regarded as biocompatible, ecologically friendly, biodegradable, and safe [26]. Nanoemulsions, nanoliposomes, nanofibers, nanohydrogels, and nanoparticles are the most common biopolymeric nanocarriers. Biopolymer-based processes have been used for antimicrobial agent nanoencapsulation as biopolymers have low toxicity and several functional roles, such as antimicrobial and antioxidant properties, gelling, emulsification, and foaming properties [1]. Other properties of biopolymeric nanocarriers include lower GRASS material content, improved encapsulation efficiency, controlled release, enabling intestinal absorption of loaded EOs, and targeted delivery [26]. These properties offer a safe and efficient delivery system and enable easy handling of chemical alteration [1].

Milk proteins, zein, soy, and gelatine are the most commonly used proteins in the preparation of biopolymeric nanocarriers. β -casein and β-lactoglobulin are two examples of milk proteins [1]. Protein-based nanoparticles are frequently produced using methods such as pH variation, phase separation, nano-spray drying, milling, rapid laminar jet, and polymer chain collapse [1]. Food-grade biopolymers, such as carbohydrates, lipids, proteins, and other biodegradable polymers, are commonly used as a wall material or coating agent in the food industry [26]. Polysaccharides, which include chitosan, alginate, starches, cellulose, pectin, and gums, are among the biopolymers that are widely used because they are abundant in nature and inexpensive. They are also safe, non-reactogenic, convenient to use, and have high biodegradability and biocompatibility [26].

### 4.6. Equipment-Based Nanocarriers

Equipment-based procedures are common industrial nanoencapsulation technologies that use specialized equipment such as a nano-spray dryer, electro-spinning, and electro-spraying to produce various types of antimicrobial-loaded nanocarriers [1]. The main advantages of equipment-based nanocarriers include industrial-scale production and rapid processing, with many advantages such as high specific surface area, high molecular orientation, high porosity, and mechanical properties.

Electrospinning has been one of the most popular, novel methods for producing antimicrobial compounds, with essential components including a high voltage supply, a feeding pump, a capillary tube, and a metal collecting screen [1]. Electrospinning has many advantages such as high specific surface area, high molecular orientation, high porosity, and mechanical properties [61]. It is a convenient method for producing nanofibers from polymer solutions with diameters varying from 2 nm to many micrometres [62]. A number of these ultrathin nanofibers include polymers, ceramics, and composites [62]. In this process, the nanofibers are affected by voltage, distance, form, and collector speed [63].

The biopolymers are guided into a capillary tube at a constant rate by the syringe pump. An electric field is generated by the specific voltage between the tip of the needle and the collector. When the voltage is increased, electrostatic repulsion becomes stronger than surface tension, causing the liquid meniscus to form into a conical shape known as the Taylor cone. Next, the liquid jet is emitted to a collector [26].

Some main types of electrospinning methods include physical adsorption, covalent immobilization, blend electrospinning, and coaxial electrospinning [26]. In the physical adsorption method, bioactive compounds are bound to the surface of obtained nanofibers by immersing the nanofibers in a bioactive compound solution [26]. The blend electrospinning process yields controlled release nanofibers with bioactive compounds dispersed throughout the matrix of nanofibers. The coaxial electrospinning method creates core antimicrobial shell nanofibers by injecting biopolymer solution and antimicrobial solution into two different syringes [26]. Tissue scaffolds, smart clothing, filtration, electrodes, pharmaceuticals, sensors, and environmental engineering can all benefit from electrospinning [63].

### 4.7. Nanoclay Nanocarriers

Nanoclays present a characteristic platelet form, soft flaky structure, low specific gravity, and a high aspect ratio with nanoscale thickness. However, nanoclays are usually incorporated with bio-based and synthetic polymers to produce a nanocomposite with these enhanced characteristics, suitable for a packaging material [64]. Reinforcing the nanoclays in polymer matrices effectively enhances properties such as the UV barrier, antioxidant, and antimicrobial activities. The incorporation of nanoclays is dependent on multiple factors such as the type of polymer/nanoclay, processing technique, the loading content, desired properties, and applications, although the organization of the nanoclay platelets in the polymer matrix is considered to be the most crucial for successful reinforcement [65]. Nanoclay-based films and coatings are advanced technologies that have been developed with the incorporation of active or functional material in terms of food packaging to extend the shelf life and maintain the quality of foodstuffs [66].

Nanoclays have been of interest for their use as encapsulants for application in active packaging with EOs, which enhances the antimicrobial and antioxidant activities, mechanical and thermal properties, and the UV barrier of the film/coating, and allows for a delivered and controlled system [67]. Thyme oil, thymol, and carvacrol have been prepared with montmorillonite clay and are promising nanomaterials for functional purposes; they encapsulate active agents or materials and can be further developed to control the release of active compounds in packaging systems [68].

## 5. Migration of EOs from Active Packaging Incorporated with Nano-Carriers

Active packaging is a type of novel food packaging system that combats several environmental factors to extend the shelf life, quality, and safety of food products [26]. EOs’ role as antioxidants and antimicrobial agents has been successfully demonstrated in many studies. However, direct incorporation into film-forming solutions raises significant concerns, such as poor miscibility, and phase separation, as well as a negative effect on film transparency [26]. Active functions that are key requirements of an active substance for incorporation into a packaging system include antimicrobial and antioxidant activity, oxygen and ethylene scavenging, and carbon dioxide emitting. Many available EOs acquire all these properties; however, they are not all suitable for food packaging applications due to their migration properties [3].

The most significant issue with the direct addition of EOs into active packaging is the migration of active compounds, which further reduces the effectiveness of active packaging during the shelf life of food products [26]. This problem can also make it difficult to produce suitable packaging for specific foods, as the active compounds in EOs can migrate into the food products and cause toxicity [3]. Biopolymeric nanocarriers containing EOs can resolve these barriers related to the direct incorporation of EOs into active packaging because they have a higher surface/volume ratio than their bulk state [26]. Many internal and external factors affect the migration of substances from packaging into the food, as not only the specific active compound can migrate, but also parameters such as solubility and temperature can have a major influence on migration in packaging; if the temperature is raised it can cause the substance to behave irregularly, and increase the movement of the molecules within the substance [27].

In active packaging systems, where active agents such as carbon dioxide emitters, oxygen and ethylene scavengers, and antioxidant antimicrobial constituents supply functions to food packaging, the interaction between packaging and food is favoured [3]. Food packaging materials that contain active compounds such as EOs can help protect against various types of degradation, as well as vapours and gases [3]. Migration is the mass transfer of the substances of the material intended to come in contact with food. It relates to the procedure of distributing substances into the food product [27]. If the component has a molecular mass lower than 1000 Da, it is very dangerous, as it is physiologically active [27]. The concentration of migration of a compound from a plastic food contact material into a food product is determined by numerous factors. These factors include time, temperature, migrant concentration in the plastic, polymer type, and food physiochemical properties [27]. For example, certain components of a food product, most notably fats and moisture content, can raise the phenolic compounds transferred from active packaging to the food product [3]. Migration tests can be used to establish the movement of active compounds in polymeric matrices under specific time and temperature conditions, based on the type of food being packaged, its usage, and storage attributes [27]. Many other characteristics are considered when conducting these migration tests such as the type of polymer [27]. A method which can be used to measure the migration of an active substance includes a chromatographic method. This method is known to identify, separate, and quantify bioactive compounds in packaging [3].

## 6. Legal Aspects of the Use of Nanocarrier Active Agents in Food Packaging

Active packaging presents many new challenges when compared to conventional packaging due to its interaction with the environment as well as the food product. The most dangerous threat posed by this food packaging is the migration of packaging ingredients into the food, as well as the inability to correctly carry out the active packaging procedure, both of which can have negative effects on the food product [32]. Each system must comply with legislation to be sold on the European market. The non-active component, such as the packaging of the active constituents, must adhere to the applicable food contact legislation [32]. The Framework Regulation (EC) 1935/2004 applies to all food contact materials. There are some specific Directives under this regulation, such as the Plastics Directive 2002/72/EC. This Directive applies to all plastic food contact materials. Numerous active packaging systems are made of various materials and are not covered by Directive 2002/72/EC, and these materials must adhere to national legislation in some countries, such as Germany and the Netherlands [32]. Other countries should accept food contact materials developed in these countries under the mutual recognition principle if such a country does not have specific regulations in the relevant fields [32].

Article 4 of the Framework Regulation specifies provisions for active materials. The requirements include the following: Changes in the composition of food should be brought about by active materials, provided that the changes comply with the community or national provisions appropriate to the food. Substances released from active packaging must be authorised and used in line with the applicable community provisions relevant to food; active packaging should not cause changes in the composition or sensory properties of the food product such as masking the spoilage which misleads the consumer. The packaging must have sufficient labelling indicating that the materials are active [32]. According to Article 15 of the Framework Regulation, the consumer and food packer must be made aware of how to use active packaging/materials safely and appropriately. EOs must be registered with the European Commission (EC) before they can be produced and used as flavouring agents in and on foodstuffs [69].

The European Commission’s Regulation (EC) No. 1334/2008 specifies a number of standards for the safe use of flavourings, as well as a list of these flavourings [69]. For example, Regulation (EC) No. 1334/2008 states some of the conditions for using flavourings or food ingredients with flavouring properties in general. The European Commission’s Regulation (EC) No. 1334/2008 stated ‘Only flavourings or food ingredients containing flavouring properties that meet the following criteria may be used in or on foods: (a) they do not pose a safety risk to the consumer’s health based on the scientific evidence available, and (b) their application does not mislead the consumer’ [69]. According to Regulation (EC) No. 1334/2008, unpleasant substances should not be added to food products if they are not on the authorized EU list [69]. EOs are also capable of causing allergic reactions in some people [3]. The use of EOs can have negative health consequences, including irritation of the skin, eyes, and mucous membranes. EOs can also cause sensitivity to oils containing phenol and aldehyde groups [3]. EOs that are employed as flavouring agents fall somewhere in the middle because they are made of natural ingredients, many of which are purposefully added to foods as separate chemicals. A present standard cannot be easily applied to the safety evaluation of EOs because they are neither a direct food additive nor food in and of themselves [70].

Active packaging must adhere to the Framework Regulation (EC) 1935/2004. The main criteria of this Framework Regulation are that active materials may cause changes in the composition or characteristics of food if they comply with the EC provisions applicable to food [32]. Active packaging should not mislead the consumer, and correct labelling should be completed. Substances may be released if the food is in full compliance with the food regulation [32].

## 7. Current Application of Nano-Encapsulated EOs in Active Food Packaging

EOs are commonly used in the food industry due to their natural antimicrobial and antioxidant properties, which help to extend the shelf life of foods [3]. Fish products, meat products, milk and dairy products, bread and baked goods, and fruit and vegetables are some of the most common types of foods in which EOs are used [3].

There are some drawbacks to EOs. EOs are susceptible to environmental stresses and have an uncontrolled release, which makes incorporating free-form EOs into food packaging structures difficult [1]. This vulnerability in EOs is due to the interaction of their unstable, volatile composition and environmental factors like light, oxidation, and heating [3]. This problem has been resolved by encapsulating EOs in liposomes, polymeric particles, and solid lipid nanoparticles before using them in packaging structures [1,3].

EOs can be applied in active packaging in the form of films and coatings [3]. Increased consumer demand for safe, fresh food products with extended shelf life, as well as environmental concerns caused by non-biodegradable petroleum-based plastic packaging materials, have stimulated interest in active food packaging films/coatings [71]. Films or coatings are thin layers of materials which have the ability to inhibit mass transfer (such as water vapour, oxygen, solute, and carbon dioxide) throughout the food as well as its environment [71]. Films are free-standing thin layers/sheets of active material. The films are typically manufactured ahead of time and can be used as packaging, covers, wrappers, and layer separation for a variety of foods. Coatings, on the other hand, are thin films that can be applied to the surface of a food product [3,71].

There are numerous examples of EOs being incorporated into active films/coatings [3]. One example of the incorporation of EOs in the film is a study based on the incorporation of cinnamon EO-loaded chitosan nanoparticles into low-density polyethylene films. The films which were produced were then used as packaging materials for enclosing fresh pork to improve the various physicochemical characteristics and quality of fresh pork throughout a 15-day storage period at 4 °C [72] (Table 2). Chitosan nanoparticles loaded with cinnamon EO with three sizes of 112 nm, 215 nm, and 527 nm were set up by ionic gelification reaction [72]. It was discovered that when compared to the other treatments, active films incorporating cinnamon EO-loaded chitosan NPs (527 nm) demonstrated a significant reduction in microbial growth, and the peroxide value, 2-thiobarbituric acid, pH, and sensory scores of the wrapped pork [72].

Another study was carried out on the quality characteristics of grapes with the use of chitosan and alginate beads containing clove EO. The freshness, firmness, flavour, odour, and overall acceptability of grapes were greatly maintained by using chitosan and alginate beads containing clove oil [93]. The encapsulated carvacrol in cyclodextrin/sodium alginate films was prepared by [94], and its antifungal properties were investigated in white mushrooms stored at 4 °C.

A recent study was carried out on developing an active internal coating for food packaging applications by using emulsion electrospinning [95]. The antioxidant molecule β-carotene was electro-spun onto a polyhydroxy butyrate-co-valerate (PHB92/PHV8) film after being encapsulated in a mixture of soy protein isolate (SPI) and polyvinyl alcohol (PVA) [95]. It was discovered that heat treatment slowed and prolonged the release of specific bioactive compounds [48].

Another study on the chemical composition of *Thymbra capitata* EO (TCEO) and its antimicrobial activity when applied with heat either as a suspension (s-TCEO) or loaded in self-assembled zein nanoparticles (zn-TCEO) was conducted [49]. Zein, a plant protein derived from corn and maize, has been proposed as a natural and biocompatible carrier. The antibacterial activity of two TCEO formulations (s-TCEO and zn-TCEO) against *Escherichia coli* O157:H7 was compared: *Listeria monocytogenes* and H7 Sakai EGD-e [49]. The bacteriostatic activity of zn-TCEO was higher than that of s-TCEO, most likely due to better dispersion in the growth media. However, zn-TCEO had lower bactericidal activity than s-TCEO, which was most likely due to the progressive release of the EO [49]. It was discovered that the combination of TCEO and heat (53 °C) had useful synergistic lethal effects, leading to the death of up to 5 log 10 cycles from both microorganisms. The effectiveness of zn-TCEO was enhanced at pH 4.0. As a result, the use of this new delivery method could be a beneficial alternative for food [49].

## 8. Conclusions

Food products are vitally protected by food packaging from environmental elements such as UV light, oxygen, water vapor, pressure, and heat. By protecting against contaminating chemicals and microorganisms, it also contributes to enhancing food safety and extending shelf life. Due to rising consumer demand for natural products and safe food, as well as the implementation of increasingly strict requirements to prevent foodborne infectious diseases, food preservation, quality maintenance, and safety are becoming increasingly important problems in the food sector. EOs are flammable liquids that are derived from various aromatic plant components and may be recognised by the fragrance molecules they contain. These bioactive substances can inhibit the growth of food-borne diseases and maintain food products, making them suitable for active packaging. Due to the fact that active components in EOs can migrate into food products and induce toxicity, it can be challenging to produce adequate packaging for particular meals. Because they have a larger surface-to-volume ratio than their bulk state, biopolymeric nanocarriers containing EOs can overcome these obstacles to the direct inclusion of EOs into active packaging. The development of biopolymeric-based active packaging that incorporates released active compounds-loaded nanocarriers is a viable food packaging method. By increasing physical, mechanical, and chemical qualities, active compound-loaded nanocarriers can extend the shelf life and durability of biopolymeric-based active packaging. Future studies should consider the deployment of active packaging with the potential to improve food safety. The benefits of the main classes of nanocarriers carrying active compounds utilized for producing sustainable biopolymeric-based active packaging with antibacterial and antioxidant capabilities should be examined.

## Figures and Tables

**Table 1:** Nanoencapsulation systems based on biopolymers for antimicrobial agents.

Nanocarrier	Antimicrobial Agent	Main Results	References
Nanoliposomes (soybean lecithin)	Nisin	Nisin produced antimicrobial and inhibitory properties against *L. Monocytogenes* and demonstrated controlled release when embedded in a HPMC matrix.	[35]
Nanoliposomes (chitosan and whey proteins)	Garlic EO	Garlic oil in an active film retarded lipid oxidation and microbial growth.	[37]
Cyclodextrin	Mustard essential oil	Mustard oil incorporated with β-cyclodextrin had shown antimicrobial activity against several microorganisms, indicating slow release in cellulose film.	[46]
Cyclodextrin	Tea Tree essential oil	Tea tree oil incorporated with β-cyclodextrin exhibited antimicrobial properties against *E. coli* which was enhanced with plasma treatment.	[47]
SLN (hydrogenated palm oil) and NLC (refined coconut oil)	Thymol, eugenol, cinnamaldehyde essential oils	Various oils incorporated into pullulan systems demonstrated anti-fungal properties against *Alternaria* spp., *A*. *niger*, and *R. Stolonifer*. SLN anti-fungal properties proved superior when compared to NLC in this experiment. NLC proved to be slightly inferior to SLN due to the higher concentration of aqueous EO at the surface.	[41]
SLN/NLC	Antioxidants (various)	Summarized the potential and application of both SLNs and NLCs in the food packaging sector with respect to antioxidant stability.	[42]
Chitosan nanoparticles	Antimicrobial peptide *temporin B*	Nano-encapsulation could increase peptide’s antibacterial activity. A sustained antibacterial action against various strains of *Staphylococcus epidermidis*.	[48]
Zein nanoparticle	*Thymbra capitata* essential oil	The antimicrobial activity of the *thymbra capitata* essential oil loaded zein nanoparticle had increased. Zein nanoparticle showed a controlled release of the essential oil.	[49]
*Chitosan*/*alginate* nanoparticles	Nisin	The nisin-loaded nanoparticles were able to inhibit more effectively the growth of *S. aureus* than free nisin during longer incubation periods in raw and pasteurized milks. The nisin-loaded nanoparticles inhibited growth more effectively than free nisin.	[50]
Chitosan/poly (ethylene oxide) nanofibers	*Cinnamaldehyde*	In time-dependent cytotoxicity studies, chitosan’s intrinsic antibacterial activity, combined with the rapid release of *Cinnamaldehyde* resulted in high inhibition rates against *Escherichia coli* and *Pseudomonas aeruginosa*.	[51]
Zein–casein nano-capsules	*Eugenol* and *thymol*	The antibacterial activities of encapsulated *eugenol* and *thymol* in milk whey was higher.	[52]
An inclusion complex’s nanofibrous webs	*Limonene*	Cyclodextrin nanofibrous containing *limonene* seemed to have a fast-dissolving property, which could improve thermal stability and shelf life. Processing antibacterial properties that could be used mainly with food.	[53]
Cyclodextrin nano-sponges	Coriander essential oil	The combination of EOs and cyclodextrin nano-sponges resulted in a controlled release of EO. Greater bacterial growth inhibition than other antimicrobial packaging.	[54]

**Table 2:** Application of nonencapsulated essential oils (EOs) in antimicrobial active packaging.

Packaging Formulation/EOs/Nanocarriers	Main Results	References
Whey protein isolate, cellulose nanofibers, rosemary oil, and TiO_2_ nanoparticles	Bio nanocomposite film displayed antibacterial and antioxidant properties such as water resistivity and enhanced tensile strength characterized through scanning electron microscopy/X-ray diffraction etc.	[73]
Chitosan based coating incorporated with nanoliposomes loaded with *Satureja khuzestanica* essential oil	The chitosan film displayed antimicrobial and antioxidant activities while the encapsulation process prolonged the release of the essential oil, overall benefiting the sensory qualities of the lamb product.	[74]
Sorbitol plasticized Whey protein isolate loaded with oregano essential oil film	The film when wrapped on beef cuts completely inhibited the growth of lactic acid bacteria as well as improving sensory attributes like reducing the total colour difference.	[75]
Modified chitosan coating loaded with red thyme, oregano, limonene, and peppermint essential oils	Chitosan coating was sprayed onto strawberries; it was concluded that it displayed inhibitory affects against moulds and microflora and allowed for controlled release.	[76]
LDPE based films loaded with linalool and methylchavicol essential oils	Methylchavicol and linalool LDPE-based films were successful at retarding and inhibiting microbial growth on cheese samples with varying parameters and influences.	[77]
Chitsoan-gelatin based coating incorporated with nano-encapsulated tarragon essential oils	Pork slices were coated in the tarragon essential oil nanoparticles and successfully inhibited microbial growth, lipid oxidation, and improved sensory attributes.	[70]
Clove essential oil loaded chitosan nanoparticles (CEO-ChNPs) to create a coating	CEO-ChNPs applied in a coating proved successful at maintaining undesired microbial, physiochemical, and sensory changes to pomegranate arils.	[78]
Soy protein isolate incorporated with carvacol to create a film	Films incorporated with carvacrol demonstrated high antimicrobial activity against *Listeria*.	[79]
Chitosan-based films loaded with eucalyptus globulus essential oil	Chitosan films containing 1.5% eucalyptus globulus oil applied to sliced sausages demonstrated high antimicrobial activity against *S. aureus*, *E. coli*, *B. cereus*, and *S. entertidis*.	[23]
Chitosan-based coating formed from nanoliposomes loaded with thyme essential oil	The coating produced with thyme EO demonstrated higher antimicrobial effects on cheese than non-encapsulated thyme oil.	[80]
Polyethylene oxide (PEO) nanofiber matrix containing β-cyclodextrins incorporated with tea tree EO	The matrix loaded with the tea tree oil displayed strong antimicrobial activity against *E. coli* when applied to beef.	[47]
Chitosan and pectin modified chrysanthemum EO nanoliposomes were created	The chrysanthemum EO loaded nanoliposomes exhibited high antibacterial activity against *C. jejuni* when applied to the surface of raw chicken.	[81]
Chitosan nanoparticles incorporated with cinnamon EO coating	Cinnamon EO proved to be successful at producing antimicrobial activity and retaining sensory attributes such as pigmentation in beef patties.	[82]
Chitosan films containing *β-cyclodextrin* inclusion with Eos	The incorporation of β-CD/EO significantly increased the antimicrobial activities of the chitosan films against *E. coli*, *S. typhimurium*, *S. aureus*, and *L. monocytogenes*.	[83]
*Origanum vulgare* L. EO-incorporated gelatin/chitosan nanoparticles composite film	The produced films showed high antimicrobial activity against four tested food pathogens including *S.* *aureus*, *L. monocytogenes*, *S. enteritidis*, and *E. coli*.	[84]
Coating structure containing chitosan nanoparticles loaded with cinnamon EO (CE-NPs)	The coating containing CE-NPs (527 nm) decreased microbial growth, peroxide value, pH (POV), 2-thiobarbituric acid (TBA), and sensory scores of the pork. The highest redness of pork samples was obtained by coating containing CE-NPs.	[71]
*Mentha pulegium* L. and *Mentha rotundifolia* EOs (L.)	The oils’ toxicity against *Rhyzopertha dominica* was determined. It was revealed that both EOs were potential chemical additive substitutes in the food and pharmaceutical industries.	[85]
Modified chitosan (MC) coating containing carvacrol nanoemulsion (CN) Hydroxyl	The loading of carvacrol nano-emulsions into modified chitosan can be used for designing an advanced bioactive coating to be entrusted on green beans, and it was active against *E. coli* and *S. typhimurium* during storage. Treating CN-coating with gamma irradiation and MAP was effective in reducing the microbial population.	[86]
Oregano Pimento	Milk protein-based edible films mixing 1% oregano, 1% pimento, or 1% oregano pimento (1:1) EO mix were added to beef muscle slices to stabilize pathogenic bacteria growth and improve shelf life while stored at 4 °C. In beef muscle samples, stabilized lipid oxidation was stabilized, whereas pimento-based films had the highest antioxidant activity. It was revealed that the film containing oregano was the most effective against both *E. coli* and *Pseudomonas* spp.	[87]
Clove essential oil	Clove EO had the most potent inhibitory activity. All microorganisms were inhibited by the clove-containing films, regardless of the film matrix or microorganism type.	[88]
Thyme essential oil	Antibacterial activity of soy protein edible films containing 5% thyme EO was tested on fresh ground beef stored at 4 °C. Antimicrobial films strongly inhibited *E. coli*, *E. coli O157:H7*, and *S. aureus* When applied to ground beef patties, coliform and *Pseudomonas* spp. counts were reduced.	[89]
Red thyme and peppermint essential oil	Red thyme and peppermint were discovered to be a powerful bioactive agent against moulds and total flora isolated from strawberries. During 14 days of storage, they were discovered to be one of the most effective preservative agents for strawberries.	[76]
Rosemary essential oil	The films containing 20% EO and intercalated with chicken breast samples had no profound impacts on the control of *psychrotrophic* or total coliform microorganisms throughout storage. The films containing 50% EO were effective in controlling coliforms during storage.	[90]
Cinnamon essential oil	The cinnamon EO content had a significant impact on the properties of the films. *P. commune* and *E. amstelodami*, fungi commonly found in bread products, were effectively inhibited by all films containing varying amounts of the EO.	[91]
Clove essential oil	Clove EO was incorporated into cassava starch films. In this study, it was found that the amount of clove essential oil required to provide films with effective antimicrobial activity against the fungi tested was too high.	[91]
Ginger essential oil	Increasing amounts of the EO modified the characteristics of the films and increased antimicrobial activity.	[92]
